# Changes in the Ultrastructure of *Candida albicans* Treated with Cationic Peptides

**DOI:** 10.3390/microorganisms8040582

**Published:** 2020-04-17

**Authors:** Alina Grigor’eva, Alevtina Bardasheva, Anastasiya Tupitsyna, Nariman Amirkhanov, Nina Tikunova, Dmitrii Pyshnyi, Maksim Kleshev, Elena Ryabchikova

**Affiliations:** Institute of Chemical Biology and Fundamental Medicine, Siberian Branch of Russian Academy of Science, Lavrent’ev av., 8, 630090 Novosibirsk, Russia; feabelit@mail.ru (A.G.); herba12@mail.ru (A.B.); aysa@ngs.ru (A.T.); nariman@niboch.nsc.ru (N.A.); tikunova@niboch.nsc.ru (N.T.); pyshnyi@niboch.nsc.ru (D.P.); max82cll@ngs.ru (M.K.)

**Keywords:** *Candida albicans*, transmission electron microscopy, cationic peptides, plasmalemma, cell wall, vacuole, cell damage

## Abstract

*Candida albicans* is becoming increasingly harmful for humans, which determines the need for new effective antifungal preparations. Currently, when testing antifungals, various morphological methods are used, among which transmission electron microscopy (TEM) is not the leading one. In this work, we used TEM to study the submicroscopic changes in *C. albicans* cells induced by cationic peptides R9F2 and (KFF)3K. Studies were performed on *C. albicans*-34 strain from the Collection of EMTC of ICBFM SB RAS in logarithmic phase. R9F2 and (KFF)3K showed an antifungal effect (MIC 10 and 20 μM) and suppressed fungal hyphal growth. Semithin and ultrathin sections of fungal suspensions incubated with 10 μM of peptides were studied at regular intervals from 15 min to 24 h. The first target of both peptides was plasmalemma, and its “alignment” was the only common morphological manifestation of their effect. Other changes in the plasmalemma and alteration of the vacuole and cell wall ultrastructure distinctly differed in cells treated with R9F2 and (KFF)3K peptides. In general, our work has shown pronounced differences of the temporal and morphologic characteristics of the effect of peptides, evidently related to their physicochemical properties. The benefit of TEM studies of ultrathin sections for understanding the mechanisms of action of antifungal drugs is shown.

## 1. Introduction

*Candida albicans*, an ordinary inhabitant of the human mucous membranes, is becoming increasingly harmful for humans, causing a local and generalized infection that requires long-term treatment. Epidemiologic data revealed the mortality rate for invasive candidiasis is 30%–40%, and the number of deaths is estimated at 1.5 million [1,2,3,4]. Worrisome prospects of the non-peaceful coexistence of humankind and fungi of the *Candida* genus, including *C. albicans*, are analyzed in many reviews that definitely substantiate the need for new antifungal drugs [5,6,7,8]. The last detailed analysis of existing antifungals pointed to the need to search for new antifungal compounds and also emphasized insufficient filling of the antifungal pipeline [9]. 

Antifungal peptides (AFPs) are a subclass of antimicrobial peptides (AMPs) and are considered promising compounds for creating effective antifungal drugs [10,11,12,13]. The main trend in the development of such drugs is the use or modification of natural peptides, primarily AMPs, which are a component of the natural defense of eukaryotes against microorganisms [14]. The antifungal effect of peptides isolated from plants [15], insects [16], fish [17], human saliva [18], chromogranin A [19], human RNAase [13], and others has been reported. 

Synthetic cell-penetrating peptides (CPPs) comprise a special group of AMPs and were initially designed for delivery of oligonucleotides into mammalian cells [20,21]. The damage caused by the CPPs to the plasma membrane, being a negative effect of their translocation into the cells, generated an interest in these peptides as antimicrobial agents. Studies showing the antimicrobial effects of a number of CPPs in relation to various microorganisms are published [22,23,24]. The use of CPPs for delivery of therapeutic molecules to cells of microorganisms, including fungi, was also examined [25,26]. The molecular composition of plasma membranes significantly differs among bacteria, mammals and fungal cells, as does the molecular composition of the cell wall of *Candida* spp. and bacteria. The authors of the review especially noted that the models of the AMP-plasma membrane interaction of mammalian and bacterial cells are “not suitable” for the analysis of the interaction of AFPs with fungal cells [11].

A rational design based on molecular characteristics of the interaction of peptide molecules with fungal cells is entering the forefront of developing new AFPs [14,27]. Obviously, the analysis of the peptide effect requires a comprehensive study of changes in microorganism cells to investigate which organoids are targets for a peptide. Indeed, a rational design operates at the level of an individual fungal cell, taking into account not only the peculiarities of its molecular organization, but also functional changes.

*C. albicans* is a small eukaryotic microorganism; its planktonic form is about 5 microns in size, which, along with pronounced pleomorphism, spherical shape, and the presence of a cell wall, complicates morphological studies of this fungus [28,29]. As a rule, researchers use phase contrast and/or fluorescence microscopy, which does not allow visualization of cell structure details. This problem is successfully solved by transmission electron microscopy (TEM) of ultrathin sections [19,30]. TEM allows bridging the gap between studying the effects of AFPs on the fungal population as a whole as well as at the level of an individual cell. The study of fine changes in cellular structures allows identifying the drug effect details on the microorganism and establishing a sequence of events unfolding in its cells. In this way, using the TEM of ultrathin sections, we revealed differences in the mechanisms of the effects of silver nanoparticles on Gram-positive and Gram-negative bacteria [31].

Peptides R9F2 and (KFF)3K were used in different studies on drug delivery, including fungal cells; however, the interaction of these peptides with *C. albicans* cells at the ultrastructural level has not been studied [23,25]. The main goal of this work was to study the development of changes in the ultrastructure of *C. albicans* cells under the influence of peptides R9F2 and (KFF)3K. Our preliminary study established that these peptides are able to inhibit the growth of some bacteria and *C. albicans* [32]. In this work, we evaluated the effect of the peptides on *C. albicans* using standard indicators of antifungal action (minimal inhibitory and fungicidal concentrations and the ability to inhibit hyphal growth). The TEM study showed that R9F2 and (KFF)3K peptides induced different changes in *C. albicans* organoids starting at different times.

## 2. Materials and Methods

### 2.1. Peptides

The peptides (KFF)3K (H2N-(Lys-Phe-Phe)3-Lys-C(O)NH2) and R9F2 (H2N-Arg9-Phe2-C(O)NH2) with >95% purity and verified by mass-spectrometry were purchased from Almabion company (Voronezh, Russia). The R9F2 amino acid sequence determines its pronounced cationic–hydrophobic properties; the cationic and hydrophobic parts are maximally spaced along the peptide chain. In the (KFF)3K peptide, the hydrophobic residues and the positive charge are uniformly alternated and distributed within the linear chain, so that linear cation-hydrophobic polarity is almost aligned. The peptides (KFF)3K and R9F2 differ in charge (+5 and +10), and the first, according to reverse-phase high-performance liquid chromatography (RP-HPLC) data, is more hydrophobic in aqueous media [32]. 

### 2.2. Microorganisms and Growth Conditions

Three strains of *C. albicans* (34, 1550, 2991), and *C. parapsilosis*, *C. tropicalis*, *C. glabrata*, and *C. (Pichia) guilliermondii* were obtained from the Collection of Extremophile Microorganisms and Type Cultures of ICBFM SB RAS (Novosibirsk, Russia). The fungi were stored at −70 °C and for the experiments, were inoculated into Sabouraud dextrose agar and cultured for 16 h at 37 °C. Then, 2 µL of this culture was sown in 100 mL of Sabouraud liquid medium and cultured in a thermostated shaker (BioSan, Riga, Latvia) at 180 rpm and 37 °C for 16 h.

We obtained a culture in the logarithmic phase according to published recommendations [33]. For this, overnight culture cells were diluted with culture medium to an optical density (OD) of 0.2 (in 200 μL) and cultured for 4 h at 180 rpm and 37 °C. The OD was measured on a flatbed reader Uniplan (Picon, Moscow, Russia) in a volume of 200 µL at a wavelength of 595 nm.

### 2.3. Minimum Inhibitory and Fungicidal Concentration Measurement

The minimum inhibitory concentration (MIC) and minimum fungicidal concentration (MFC) of R9F2 and (KFF)3K peptides were determined by serial dilution for all above listed species. Chlorhexidine, 1.0 mkM (Renewal, Novosibirsk, Russia), was used as a reference preparation. 

Cells in the middle of the logarithmic growth phase were concentrated by centrifugation; then the cell concentration was brought to ~1 × 10^5^ CFUs/mL with fresh nutrient medium. The final concentrations of the R9F2 peptide in the medium were 10, 5, 2.5, 1.25, and 0 μM, and of peptide (KFF)3K, 20, 10, 5, 2.5, and 0 μM. Concentrations of chlorhexidine were 50, 25, 12.5, 6.25, and 0 μM. Cultures of *Candida* spp. were incubated with the preparations in 96-well plates at 37 °C and 580 rpm in thermostated shaker ELMI ST-3 (SIA ELMI, Latvia) for 24 h in a volume of 200 µL, with three repetitions. Fungal growth was evaluated after 24 h by measuring of OD_595_ in each well as described earlier [34]. MIC and MFC values were determined basing on their definitions. The MIC is the lowest concentration of an antibiotic that inhibits in vitro visible growth of the fungal culture. The MFC is the lowest concentration of an antibiotic that causes the death of 99.9% of the initial number of culture cells in vitro. Finally, the minimum values were selected from three repetitions of obtained data, which are presented as MIC and MFC in Table S1.

All subsequent experiments in this study were performed using *C. albicans-34* strain (in text: *C. albicans*) in the middle of the logarithmic growth phase.

### 2.4. Effect of (KFF)3K and R9F2 Peptides on C. albicans Hyphal Development

Suspensions of *C. albicans* cells were adjusted to a concentration of ~1 × 10^2^ CFUs/mL and incubated in Spider medium in 96-well plates at 37 °C and 5% CO_2_ [17,35]. Peptide R9F2 or (KFF)3K or chlorhexidine was introduced into the culture at concentrations of 1.25, 2.5, and 5 μM and incubated for 24 h. The suspension incubated without preparations served as a negative control (intact cells). Then, hyphal growth was assessed using a Zeiss Axio Imager A2 inverted microscope (Carl Zeiss, Jena, Germany) similar to [17]; the percentage of spherical cells was determined in triplicate for each concentration of preparation (at least 100 cells were counted). 

For illustration purposes, *C. albicans* samples were fixed with 4% paraformaldehyde after 24 h of incubation with preparations, and smears were prepared, stained with Azur II, and examined under a Leica DM 2500 light microscopes (Leica, Wetzlar, Germany).

### 2.5. Processing of the Samples for Morphological Studies

Peptide R9F2 or (KFF) 3K at a final concentration of 10 μM (MIC for R9F2, Table S1) or chlorhexidine (5 μM) was added to 12 mL of *C. albicans* (~5 × 10^5^ CFUs/mL). The cells were cultured at 37 °C for 24 h; aliquots of the suspension were collected at certain intervals and centrifuged at 4000 rpm at +4 °C for 5 min. The pellets were fixed after 15, 45, 75, 105, and 135 min and 3, 4, 5, 6, 7, 8, and 24 h of incubation with the preparations. As a control, the same suspensions of *C. albicans* cells (intact cells) without preparations were used.

The study of ultrathin sections of *C. albicans* cells in TEM calls for special attention. The niceties of fungal cell processing for TEM were noted from the beginning of their studies: depending on the chemical nature of the fixative, organoids are better or worse preserved and visualized, and the cytoplasm has higher or lower electron density [36,37]. We tried different ways of fixing *C. albicans* cells and found that the most suitable was overnight fixation in 2.5% glutaraldehyde (GA) or in a mixture of 4% paraformaldehyde (PFA) and 2.5% GA (2:1). After washing from fixative, the samples were postfixed with 1% osmium tetraoxide solution for 1 h, dehydrated in ethanol and acetone according to the standard method, and then embedded in an epon-araldite mixture. All reagents for microscopic studies were purchased from EMS (Hatfield, PA, USA).

Ultrathin and semithin sections from obtained hard blocks were prepared on an ultramicrotome EM UC7 (Leica, Wetzlar, Germany) using a diamond knife (Diatome, Nidau, Switzerland). We stained semithin sections with Azur II and examined these using a Leica DM 2500 light microscope (Leica, Wetzlar, Germany). Ultrathin sections were contrasted (or not contrasted) with (2%) water solutions of uranyl acetate (UAc) and lead citrate (LC) and examined in a JEM 1400 TEM (JEOL, Japan). Digital images were collected using a Veleta side-mounted camera (EM SIS, Muenster, Germany).

## 3. Results and Discussion

### 3.1. Antifungal Activity of R9F2 and (KFF)3K Peptides Against Candida Species

Peptides R9F2 and (KFF)3K demonstrated high antifungal efficacy against all studied *Candida* species (Table S1) The MIC value for the R9F2 peptide was about 10 μM for all studied *Candida* species at a concentration of fungal cells of ~1 × 10^5^/mL. At the same time, the (KFF)3K peptide showed variations in the MIC value from 20 μM to 5 μM (Table S1). The MFC value of the R9F2 peptide for all studied Candida species was 10 or >10 μM. The (KFF)3K peptide showed an MFC value of 20 or >20 μM for all studied species, except *C. guilliermondii* (10 μM) (Table S1). In total, the MIC and MFC values of both peptides were close for each species. Chlorhexidine MFC varied noticeably (12.5–50 μM) and mostly exceeded values of both peptides for each individual Candida species (Table S1). Thus, both peptides possessed an antifungal effect in concentrations not exceeding the concentration of chlorhexidine. Earlier, we showed the absence of hemolytic and cytotoxic activity of these peptides for mammalian cells [32].

All subsequent experiments in this study were performed using *C. albicans-34* strain (in text: *C. albicans*) in the middle of the logarithmic growth phase.

The ability to inhibit the growth of hyphae is one of “usual” characteristics studied in new antifungals, since hyphae and pseudohyphae are the main form of existence of *C. albicans* in human hosts [36]. We also examined the influence of R9F2 and (KFF)3K peptides on hyphal growth. First, the inhibition of hyphal growth was evaluated microscopically in 96-well plates after 24 h incubation of *C. albicans* with 1.25, 2.5, or 5 μM of R9F2 or (KFF)3K or chlorhexidine. Peptide R9F2 inhibited hyphal growth by 70% at a concentration of 1.25 μM, while (KFF)3K reached this level of inhibition at 2.5 μM concentration (24 h of incubation). Chlorhexidine suppressed hyphal growth by 50% at a concentration of 2.5 μM. Both peptides and chlorhexidine completely inhibited hyphal growth at a concentration of 5 μM; all wells contained only spherical cells.

Images of the control and treated cultures of *C. albicans* are presented in Figure S1. The control culture was composed of long filamentous hyphae and rare rounded cells (Figure S1A). Smears of *C. albicans* treated with 2.5 μM (KFF)3K contained many clusters of spherical cells (Figure S1B); similar pictures were observed in fungal smears treated with 1.25 or 2.5 μM of R9F2 or 1.25 μM (KFF)3K (images are not shown). Formation of hyphae did not occur at a concentration of both peptides of 5 μM;, the smears showed only spherical cells (Figure S1C,D). Treatment with chlorhexidine at a concentration 2.5 μM led to the formation of clusters composed of spherical cells; many single cells were present in smears (Figure S1E). A chlorhexydine concentration of 5 μM completely blocked hyphal formation; the smears contained only spherical cells (images are not shown).

Thus, both R9F2 and (KFF) 3K peptides showed the ability to inhibit hyphal growth in *C. albicans* at concentrations similar to those of the common antiseptic chlorhexidine. Along with indicators MIC and MFC (Table S1), these data indicate a clear antifungal action of R9F2 and (KFF)3K peptides selected for this research on the *C. albicans-34* strain. 

### 3.2. Light Microscopy of C. albicans Cells Treated With R9F2 and (KFF) 3K Peptides

*C. albicans* cells in logarithmic phase actively reproduce by budding and are represented by clusters of several cells at different stages of the life cycle. Research articles are usually illustrated with images showing total views of the yeast cells in phase contrast (for example, [17,29,36,38]). Sizes, rounded shape and, especially the cell wall of *Candida* spp. cells make it impossible to study them in paraffin sections. The alternative way to examine the yeast cell morphology could be the use of semithin sections made from hard blocks embedded in epoxy resin for TEM. 

**Figure 1:**
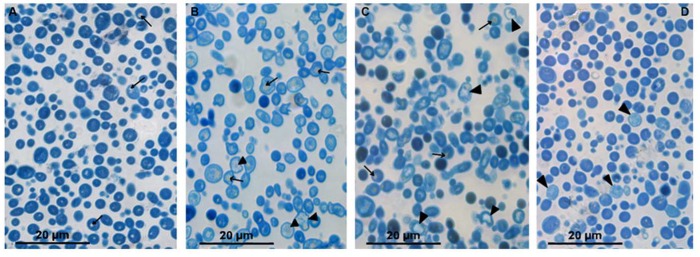
Semithin section of *C. albicans* suspensions stained with Azur II. (**A**) intact cells. Cells incubated for 4: (**B**) with (KFF)3K peptide; (**C**) with R9F2 peptide; and (**D**) with chlorhexidine. Arrows show lipid droplets; arrowheads–destroyed cells.

We conducted a study of Azure-II-stained semithin sections (about 1 μm thick) from *C. albicans* samples to determine whether such sections could provide useful information for assessing the effects of peptides R9F2 and (KFF)3K. The rounded or oval cells of *C. albicans* control culture (intact cells) in semithin sections (Figure 1A) were (3.9 ± 0.1) × (3.4 ± 0.01) μm in size and were uniformly stained in bright blue, including the cell wall. Small rounded inclusions of light yellow color (lipid droplets) were occasionally observed in the cytoplasm. These morphological characteristics of intact cells did not change during 24 h of observation (data not shown).

The half-hour incubation with peptide (KFF)3K led to a noticeable decrease in the intensity of cytoplasm staining, while the cell wall remained bright blue. The cytoplasm contained “flakes” colored in different shades of blue and small lipid droplets and colorless areas. The first colorless (dead) cells bordered with bright blue cell walls were detected after 75 min of incubation with peptide (KFF)3K; their amount increased during the incubation. High cell polymorphism was observed in *C. albicans* sample after 4 h of incubation, reflecting different degrees of cell damage by the peptide. Against the background of light-damaged cells, bright blue cells were distinguished, which did not visually differ from intact ones; their reproduction sometimes was observed (Figure 1B).

Incubation of *C. albicans* with the R9F2 peptide for 30 min did not cause as pronounced morphological changes as the peptide (KFF)3K. Both the cytoplasm and the cell wall remained bright blue and were indistinguishable. In some cells, colorless areas in cytoplasm and small lipid drops were noted. The first dead cells were registered after 75 min of incubation with peptide R9F2, and then their amount increased. After 4 h of incubation with the peptide, cells of various morphology were observed in the sections, as in the case of the (KFF)3K peptide. The cells showing morphology of intact cells were also present (Figure 1C).

Incubation with chlorhexidine led to visible morphological changes in rare *C. albicans* cells after 2 h; these cells were stained pale blue. After 4 h of incubation, the number of pale cells increased, and the cells looked somewhat swollen; small colorless areas were visible in the cytoplasm; and many cells had an intact morphology (Figure 1D). Thus, semithin sections allowed us to see differences in the morphological features of the cell response to R9F2 and (KFF)3K peptides and chlorhexidine compared to intact cells.

The peptides R9F2 and (KFF)3K influenced size and shape of *C. albicans* cells. Table S2 shows values of the morphologic index (length–width ratio, [29]), and cell length and width of intact cells and their changes during incubation with R9F2 and (KFF)3K. The changes in cell length illustrate enlargement of the fungi treated with both peptides (Table S2). Morphological indexes varied during the time of incubation with peptides; however, they differed significantly from the control value (Table S2). It should be noted that the cell boundaries on semithin sections are clear, which simplifies size measurements and increases their accuracy. The area of semithin sections makes it possible to analyze a large number of cells and evaluate the share of changed cells—it is almost impossible to make such assessment using ultrathin sections. Another advantage of semithin sections is that they come from specimens prepared for TEM studies and do not need special processing. We may conclude that examination of yeast cell semithin sections is informative and could give useful data. 

### 3.3. Ultrastructure of C. albicans Intact Cells

The ability of electron microscopy to visualize cell ultrastructure and changes in cell organoids is widely used in various studies of mammalian cells, but not in studies of fungi. To a large measure, this is related to very high density of the yeast cytoplasm, which makes it difficult and sometimes impossible to recognize cell structures after routine sample processing (fixation with aldehydes, osmium postfixation, contrasting with UAc and LC [30,39]. Researchers studying the membrane structures of *C. albicans* and other yeasts use special methods of fixation, for example, potassium permanganate, which destroys ribosomes and the cytoskeleton, making the cytoplasm “empty” [28,30]. The “enlightenment” of the cytoplasm could be achieved by treatment of cells with RNase [40] and sodium metaperiodate [41]. Obviously, such methods are poorly applicable for the analysis of experimental effects on the morphology of *C. albicans*.

We suggest a simple way allowing us to visualize the structure of both the cell wall and the cell. This way (analyzing cells without contrasting of sections with UAc and LC) does not require additional processing and allows us to analyze the same sections before and after contrasting.

**Figure 2:**
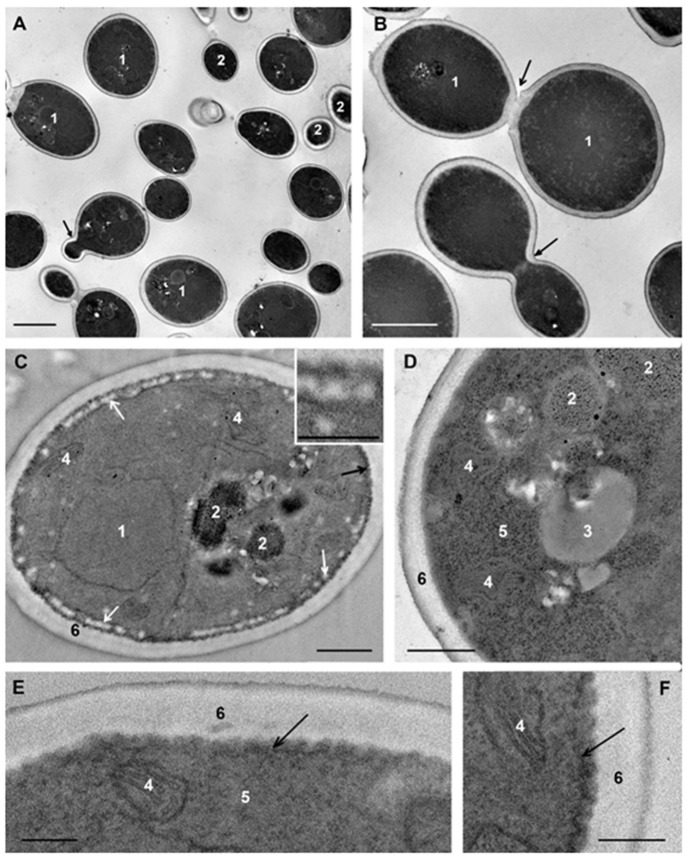
Ultrastructure of *C. albicans* intact cells. (**A**,**B**) general view of the suspension: 1–sections through the cell central part; 2–cell fragments; black arrows show budding cells. (**C**) “a tape” of “balls” at the cell periphery (shown by white arrows); the insert shows “balls” at high magnification. (**D**) cell organoids; (**E**,**F**) areas of the cells with a wavy plasma membrane (shown by black arrows). **1**–nucleus; **2**–vacuole; **3**–lipid droplet; **4**–mitochondria; **5**–cytoplasm; **6**–cell wall. (**E**,**F**) ultrathin sections without contrast. Scale bars correspond to: 2 μm (**A**,**B**), 500 nm (**C**,**D**), and 200 nm (**E**,**F**).

*C. albicans* cells that were not subjected to experimental influence (intact cells) in ultrathin sections (thickness about 70 nm) had an oval-rounded shape; pictures of budding were common. Depending on the section level, cells were represented by the central part (the cross-section size was about 4 μm), or by smaller fragments (Figure 2A,B).

The plasma membrane adheres tightly to the cell wall and this makes difficult to distinguish membrane after contrasting. Without contrasting, the membrane is visualized better; in the sections of some cells it is smooth, while in others, numerous folds (depth up to 100 nm) increase the area of the plasma membrane giving it a wavy appearance (Figure 2C–F). The same folds of the *C. albicans* plasma membrane in the logarithmic phase were described earlier [42].

The eukaryotic plasma membrane is a selective barrier that ensures the penetration of various compounds into the cell by different ways, including endocytosis. The process of clathrin-dependent endocytosis has been studied in detail in *S. cerevisiae* cells [43]. Similar studies of *C. albicans* have not been conducted; researchers usually confine themselves to the fact that fluorescent labels enter cells and migrate into vacuoles and call this process “endocytosis” [38,44,45]. This partly could be related to the active study of another very important process: endocytosis of *C. albicans* by host cells. *C. albicans* is able to induce this process, providing invasion of the fungus in the host tissue, and this ability is a factor of its virulence and pathogenicity [45,46,47].

At the periphery of many cells, spherical structures (60–100 nm) composed of granular material of low electron density without a limiting membrane appeared as small uniform balls were observed. These “balls” often composed a “tape” encircling the cell (Figure 2C); sometimes they formed large aggregations. The “balls” are seen in illustrations in many articles; however, the authors do not pay attention to their identification. We suppose that the “balls” could be a kind of “reserve deposit” in a cell preparing for division and growth.

The nucleus of intact *C. albicans* cells (Figure 2C,E) was mostly irregular in shape and located in the cell center, or may be displaced to the cell wall. The nucleus envelope consisted of two membranes, visible in cross sections, and the nucleoplasm was homogeneously fine-grained. The observed nucleus morphology corresponds to the published data [48]. The cytoplasm of *C. albicans* cells contains a few large mitochondria with transverse cristae (Figure 2C–E). The Golgi apparatus could be seen after “enlightenment” of the cytoplasm [41], so this structure did not appear in images in our study. Homogeneous lipid droplets of average and sometimes high electron density were common structures in *C. albicans* cells (Figure 2D). They had various sizes and were often in close contact with the vacuole.

In our study, vacuoles of intact *C. albicans* cells were mainly represented by several non-large structures of high electron density (Figure 2C); such structures were previously described in intact *C. albicans* cells at the logarithmic growth phase [38,49,50,51]. Another variant of the vacuole was non-large structures of medium electron density containing electron-dense particles (Figure 2E). The vacuole membrane was clearly visible only in perpendicular sections, similar to other membranes of fungal cells. Vacuoles were present but not in all sections of intact cells, and the presence was the main element determining the polymorphism of *C. albicans* cells in a TEM study of ultrathin sections.

### 3.4. Changes of Cell Wall Ultrastructure under the Influence of R9F2 and (KFF)3K Peptides

Ultrathin sections can pass a cell at different angles to the plane of membranes and the cell wall, thereby making the membranes “invisible”, and the wall structure and its dimensions distorted. The need to select strictly perpendicular sections for studies of the structure and measurements of the cell wall was noted more than 30 years ago; the author also recommended avoiding zones of bud scars, in which the wall thickness may differ [52].

The cell wall of *C. albicans*, similar to other fungi, is a natural barrier to any substances on the way to a cell. The fungal cell wall is layered, and details of its molecular structure are described in comprehensive reviews [11,53]. In *Candida* and *Saccharomyces* species, the outer layer of the cell wall comprises highly mannosylated glycoproteins and covers the inner layer, composed mainly of β-(1,6) glucans and β-(1,3) glucans. Fungal cell walls also contain a large number of various molecules responsible for regulating the synthesis of wall components, signaling, and transport. The cell wall is considered as a complex structure that provides many critical vital processes in fungi [53]. Cryoelectron microscopy made it possible to visualize some components of cell walls, including mannoprotein fibrils [53,54]. It is interesting that cell walls mainly consist of molecules that are absent in the human body, which determines interest in it as a target for drugs [11,52,53].

**Figure 3:**
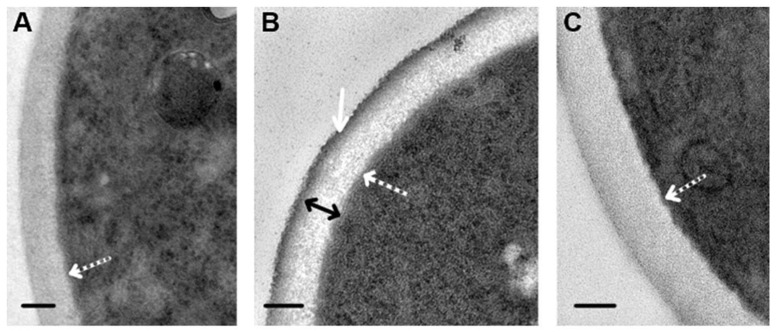
Ultrastructure of cell walls in intact *C. albicans* cells: (**A**) fixation in 2.5% GA, contrasting with UAc and LC. (**B**) fixation in a mixture of PFA and GA, contrasting with UAc and LC. (**C**) fixation in a mixture of PFA and GA, without contrasting. White dotted arrows show the plasma membrane; the white arrow shows the outer electron-dense layer; the two-way arrow shows the inner layer of the cell wall. Scale bars correspond to 200 nm. Ultrathin sections.

Our study revealed that visibility in the TEM of cell walls depends on the composition of the fixative solution. Fixation in GA resulted in homogeneous cell walls, despite contrasting of sections with UAc and LC (Figure 3A). When fixed in a mixture of GA and PFA, the wall structure in contrasted sections was detailed (Figure 3B): the outer electron-dense layer about 20 nm thick (corresponding to the mannosylated glycoproteins layer) and the inner layer (about 100 nm) formed by an electron-transparent matrix containing variously oriented filamentous structures were clearly visible. In the absence of contrasting, cell walls in the same section had a low electron density and were homogeneous, and the outer electron-dense layer was poorly distinguishable; however, it formed a clear wall boundary, and the fibers of the inner layer were not visualized (Figure 3C). The thickness of the cell wall in perpendicular sections through the cell central part was 126.3 ± 10.53 nm. Thus, the method of fixation and contrasting influences the appearance of cell walls seen in TEM.

**Figure 4:**
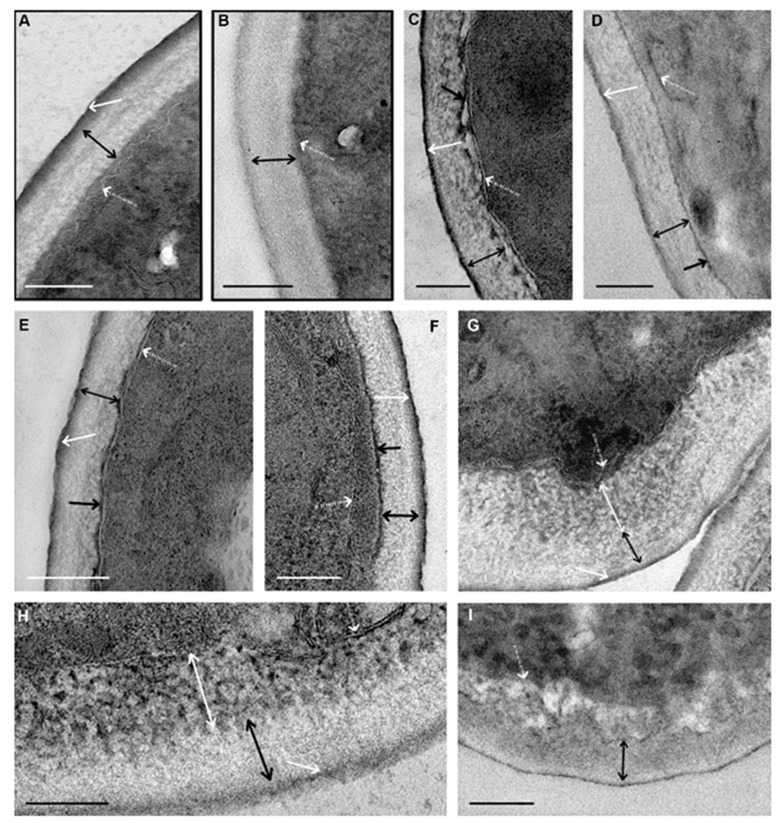
Changes in the ultrastructure of cell walls of *C. albicans* at the greatest severity when exposed to the peptides (KFF)3K (**C**–**F**, 2 h incubation) and R9F2 (**G**–**I**, 6 h incubation). For comparison: (**A**,**B**) cell wall of the yeast intact cells (framed). White dotted arrows show the plasma membrane; white arrows–wall outer electron-dense layer; black two-way arrows–wall inner layer; black arrows show the layer of electron-dense substances on the wall inner surface; white two-way arrows show fibrillar structures. (**B**,**D**) sections without contrasting. Scale bars correspond to 200 nm.

We observed clear differences in cell wall changes and their starting time during the incubation with peptides. Figure 4 shows images of cell walls at the greatest severity of changes induced by each of the peptides. In these images, the reader can see the appearance of different structures mentioned in the text.

The first changes caused by peptide (KFF)3K in the cell wall ultrastructure were observed after 30 min incubation with *C. albicans* suspension. Small pieces of a homogeneous electron-dense substance appeared on the inner side of the wall (Figure 4C–F). As incubation with the peptide progressed, the thickness and length of these pieces increased, and the substance gradually covered the entire surface of the cell. The substance was visualized without contrasting; this means that it binds to osmium tetroxide (osmiophilia). It can be assumed that this substance (1) is a component of the inner layer of the wall chemically altered by the (KFF)3K peptide or (2) reflects the accumulation of the osmiophilic substance in the wall during its increased migration to or from the cell.

After 45 min of incubation with the (KFF)3K peptide, single fibers of medium electron density were detected in the wall inner layer, and their contrast and number gradually increased, and after 135 min of incubation, distinct electron-dense fibers were observed (Figure 4C). Thus, peptide (KFF)3K caused disorganization of cell walls of *C. albicans*, at the same time widening of the space between the cell wall and plasma membrane, and the wall was detached from the cell (Figure 4C,D). Amorphous material of average electron density, electron-dense particles, and membrane fragments could present in this space. Described alterations of cell walls were observed in sections of almost all yeast cells after 135 min of incubation with peptide (KFF)3K. Incubation of *C. albicans* with the peptide for 6 h resulted in deformation and loosening of cell walls, which thickened to 200 nm (Figure S2).

Morphological changes of the cell wall (Figure 4G–I) induced by the peptide R9F2 were registered 45 min later than in the case of peptide (KFF)3K. Thinning of the outer electron-dense layer, loosening, and thickening of the inner layer were observed in wall sections. The thinning indicates peptide R9F2 interaction with components of the outer layer (mannosylated glycoproteins); this effect was absent under incubation with peptide (KFF)3K. Wall changes caused by the R9F2 peptide progressively increased during 6 h of incubation: the outer electron-dense layer gradually disappeared; the thickness of the inner layer increased up to 200 nm. Electron-dense “branches” and “strings” become visible in the inner layer, anastomosing among themselves with the formation of a “network”; these changes were most clearly visible in cell sections after 6 h of incubation with the peptide (Figure 4F,G).

A comparison of the nature and dynamics of ultrastructural changes in *C. albicans* cell walls under the influence of peptides (KFF)3K and R9F2 shows clear differences. Most significant, in our view, are the more rapid effect of peptide (KFF)3K; the occurrence of electron-dense material at the border of the cell wall under the action of peptide (KFF)3K; the disappearance of the cell wall outer layer, loosening and structuring of wall inner layer components under the action of peptide R9F2. Therefore, peptides (KFF)3K and R9F2 interact differently with cell walls of *C. albicans*, and morphological signs of this interaction are registered much later than the changes in the plasma membrane described below, although peptide molecules must overcome the cell wall to reach the plasma membrane.

### 3.5. Impact of R9F2 and (KFF)3K Peptides on C. albicans Cell Ultrastructure

The plasma membrane of *C. albicans* cells serves as a selective barrier, ensuring metabolism in fungal cells and, according to the published data, differs in molecular composition from plasma membranes of bacteria and mammals; moreover, it differs even from the membrane of a close “relative” *S. cerevisiae* [11]. The results of published studies reported that the plasma membrane of *C. albicans* is the first target for AFP [16,27].

To identify differences between effects of peptides (KFF)3K and R9F2 on *C. albicans* cells, it was important to study initial steps of the interaction. The need to wash cells from the culture medium determined the duration of the minimal incubation period of cells with peptides—15 min.

**Figure 5:**
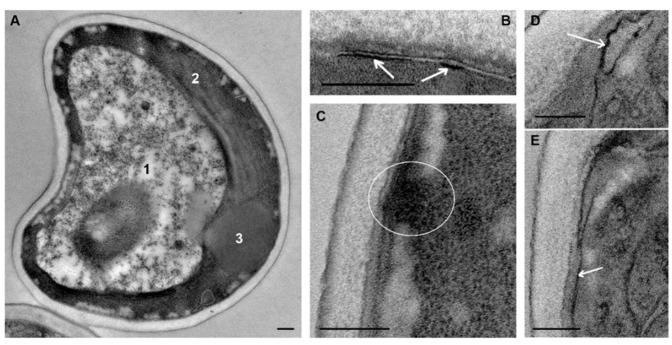
Changes in *C. albicans* cell ultrastructure after 15 min incubation with peptide (KFF)3K. (**A**) large vacuole of irregular shape (**1**) in the cytoplasm (**2**); 3–a lipid droplet; electron-transparent “balls” are seen along the periphery of the cell. (**B**–**E**) osmiophilic areas in the plasma membrane (shown by arrows). (**C**) a “bridge” (shown by an oval) is visible between the cytoplasm and osmiophilic site of the plasma membrane. (**B**–**E**) sections without contrasting. Scale bars correspond to 200 nm.

The first change in *C. albicans* ultrastructure under the influence of (KFF)3K peptide was detected 15 min after peptide addition to the culture medium and involved the vacuole and plasma membrane. The vacuole increased in size, often causing cell deformation; its cavity was filled with an electron transparent material in which electron-dense particles were dispersed (Figure 5A). It is interesting that the peptide (KFF)3K within 15 min or less caused changes in the vacuole, which is a cytoplasmic organoid. Decrease in vacuole electron density (Figure 5A) compared to intact cells (Figure 2) suggests a disturbance of ion transport through the vacuole membrane and the flow of water into the vacuole cavity, the membrane of which carries numerous transport channels [49,50,55,56,57,58].

Obviously, damage to the vacuole structure could not occur without alteration of the plasma membrane, which first came into contact with peptide (KFF)3K. Within 15 min, the membrane lost its folding, and this “alignment” was noticeable both in contrasted and non-contrasted sections. Small electron-dense areas appeared in the plasma membrane (Figure 5B–E), the number and length of which increased during subsequent incubation with the peptide. These areas undoubtedly reflect damage to the membrane, and their independence on contrasting gives a reason to consider them as osmiophilic. Figure 5C shows an electron-dense “bridge” between the damaged area of the plasma membrane and the cytoplasm, probably reflecting the formation of a hole in membrane through which cell contents can outflow into a space between the cell and cell wall. The “balls” of low electron density were observed in sections of many cells including those with damaged plasma membranes (Figure 5A,C).

**Figure 6:**
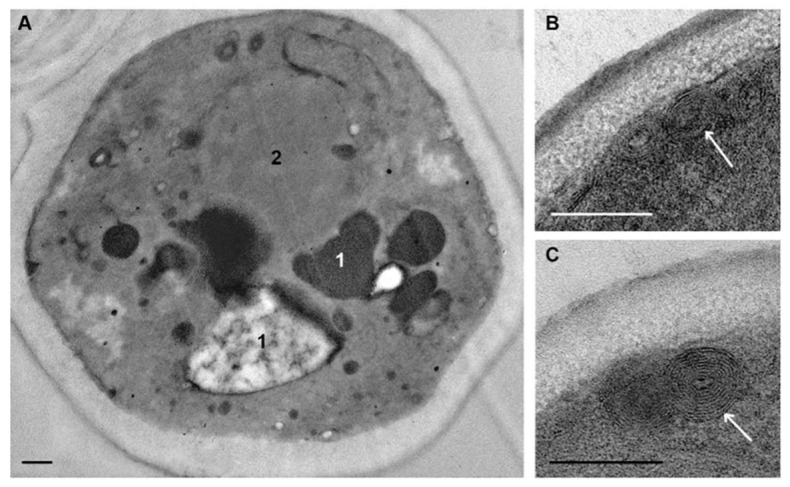
Changes in *C. albicans* cell ultrastructure after 30 min incubation with peptide (KFF)3K. (**A**) cell showing polymorphisms of the vacuole (**1**); **2**–nucleus. (**B**,**C**) multilayer structures (shown by arrows) on the border of the plasma membrane and the cell wall. (**A**,**C**) sections without contrasting. Scale bars correspond to 500 nm (**A**) and 200 nm.

Incubation of *C. albicans* with peptide (KFF)3K for 30 min led to distinct changes in the cells compared to 15 min incubation. Vacuoles acquired a bizarre shape; their protrusions extended throughout cytoplasm, often contacting with lipid droplets, and the electron-transparent content was observed only in some cells; mainly vacuoles contained granular and amorphous material of high and/or medium electron density (Figure 6A). Vacuole changes were better visualized without contrasting, as well as osmiophilic areas in the plasma membrane, the length of which increased. Formation of multilayer flattened structures was observed in areas of visually intact plasma membranes (Figure 6B,C). The number of layers in these structures varied; sometimes they contained amorphous material, which was apparently a piece of cytoplasm (Figure 6B). Analysis of sections shows that these multilayer structures were subsequently located in the space between the plasma membrane and cell wall. Apparently, in this way, the cell got rid of membrane portions that were less damaged than in osmiophilic sites. This was also evidenced by a lack of noticeable osmiophilia of the multilayer structures; obviously, the mechanism of their formation differs from the formation of osmiophilic sites in plasma membranes.

**Figure 7:**
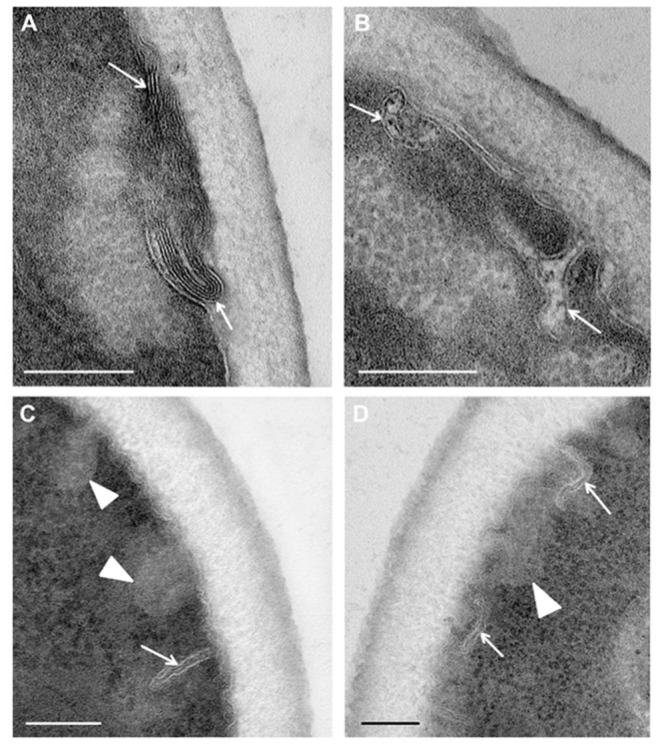
Changes in the ultrastructure of the plasma membrane of *C. albicans* cells incubated for 30 min with the R9F2 peptide. (**A**) a multilayer structure (shown by arrows) formed by the plasma membrane; branched (**B**) and narrow (**C**,**D**) invaginations of the plasma membrane extending into the cytoplasm (shown by arrows). Clusters of thin-membrane structures resembling foam (shown by the arrowhead). (**C**,**D**) sections without staining. Scale bars correspond to 200 nm.

Study of ultrathin sections of *C. albicans* cells, incubated for 15 min with R9F2 peptide at the same concentration as (KFF)3K, did not reveal signs of cell alteration; the cell morphology did not differ from intact cells described above. Changes in plasma membranes were detected only after 30 min of incubation with the R9F2 peptide; at the same time, visible changes in other cellular structures were not found. We observed alignment of plasma membranes in most cells, the formation of multilayer folds (Figure 7A), branched and narrow invaginations protruding into the cytoplasm (Figure 7B,C), as well as structures resembling foam and formed by thin membranes (Figure 7D). Obviously, all these structures reflect damage to the plasma membrane by the R9F2 peptide, and their diversity suggests the interaction of the peptide with different components of the plasma membrane. It should be emphasized that there was no formation of osmiophilic sites on plasma membranes when exposed to peptide R9F2.

The observed ultrastructural changes of the *C. albicans* plasma membrane in response to peptides (KFF)3K and R9F2 indicate different interactions of peptides with membrane components. The peptide (KFF)3K cause a destructive effect on a number of membrane components, resulting in the appearance of osmiophilic sites and holes. In addition, other areas of the plasma membrane formed multilayer structures without signs of osmiophilia, and dynamics of their changes indicate rejection of these structures by the cell. It could be proposed that formation of multilayer structures represent a lesser extent of membrane damage by the peptide (KFF)3K. Thus, the plasma membrane of *C. albicans* treated with the (KFF)3K peptide show three morphological conditions: visually intact areas, multilayer structures, and osmiophilic sites and holes. Perhaps these features are due to the different molecular composition of the plasma membrane of *C. albicans* cells, which is at various stages of the life cycle in the logarithmic growth phase.

The peptide R9F2 also led to various changes in ultrastructure of the plasma membrane of *C. albicans*, but their character was clearly different from those of peptide (KFF)3K. It can be supposed that formation of multilayer folds and a thin-membrane “foam” occurs similarly to the effect of polyarginine peptides on the plasma membrane of mammalian cells, where the membrane bilayer splits and loosens, and its layers are inverted [59,60].

**Figure 8:**
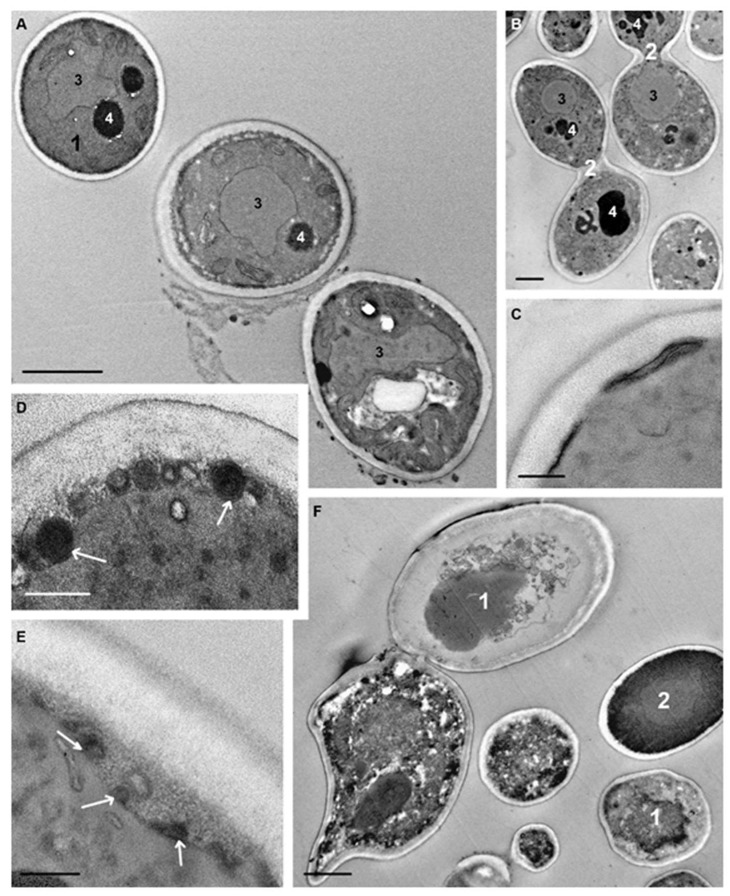
Changes in ultrastructure of *C. albicans* incubated for 75 min with peptide (KFF)3K. (**A**,**B**) diversity of cell morphological changes: **1**–a cell with minimal destructive changes; **2**–budding cells; **3**–nucleus; **4**–vacuole. (**C**) osmiophilic material on the cell surface. (**D**) multilayer “balls” on the border of the plasma membrane and the cell wall (shown by arrows). (**E**) membrane “balls” in the expanded space between the cell wall and the cell. (**F**) necrotic (**1**) and “normal” (**2**) *C. albicans* cells after 6 h of incubation with peptide (KFF)3K. (**A**–**E**) sections without contrasting. Scale bars correspond to 1 μm (**A**,**B**,**F**) and 200 nm (**C**–**E**).

Incubation of *C. albicans* cells with peptide (KFF)3K for 75 min resulted in a very high diversity of cell morphology due to the varying degree of destructive changes (Figure 8A,B). In order not to “drown” in morphological details, we focused on changes in those cellular structures that were the first targets for the peptide. The vacuoles had a diverse shape and basically granular contents; there were some vacuoles with electron-transparent and homogeneous electron-dense contents, and vacuoles of many cells were of a mixed type (Figure 8A,B).

Osmiophilic sites remained in the plasma membrane, and accumulation of osmiophilic material associated with the plasma membrane was observed (Figure 8C). The multilayer structures noted above were still present in space between the plasma membrane and cell wall. Expansion of this space (Figure 8D,E) was a noticeable change in the structure of *C. albicans* cells and was observed in many cells. Judging by the changes in contents during observations, at first an unstructured granular material appeared in the space, apparently being an exudate of the cytoplasm. Later, membrane structures and various particles appeared in the space that could enter it upon rupture of the plasma membrane due to damage by peptide (KFF)3K.

**Figure 9:**
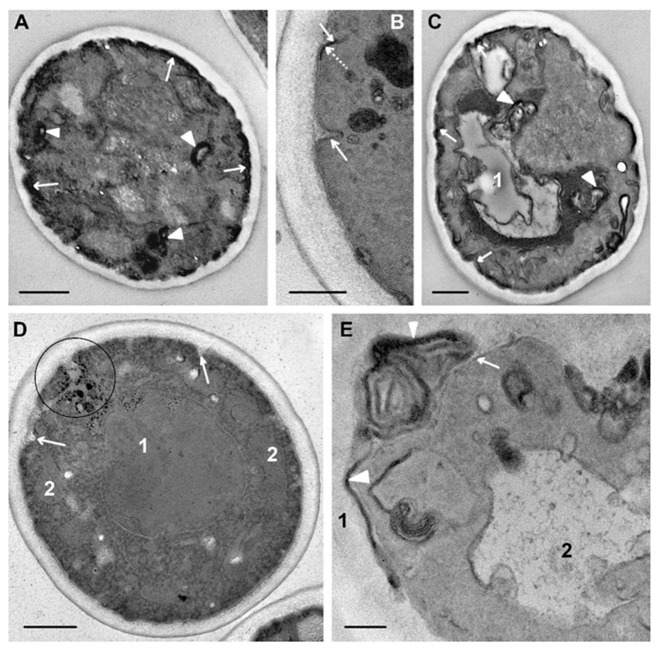
Changes in ultrastructure of *C. albicans* incubated for 75 min with R9F2 peptide. (**A**) a cell with destroyed organoids, arrows show osmiophilic material on the cell surface; arrowheads show osmiophilic multimembrane structures in the cytoplasm. (**B**) plasma membrane invaginations (shown with arrows) at higher magnification; dotted arrows are pointed at osmiophilic areas. (**C**) a cell with an electron-transparent vacuole (**1**) and destroyed organoids; the arrows show osmiophilic material on the cell surface; arrowheads show osmiophilic multimembrane structures in the cytoplasm. (**D**) slightly changed cell: **1**–nucleus; **2**–mitochondria; arrows show plasma membrane invaginations; oval shows deep branched invagination. (**E**) a part of a damaged cell. Arrow shows plasma membrane; arrowheads show osmiophilic membrane structures on the cell surface; 1–cell wall; 2–vacuole. (**E**) unstained section. Scale bars correspond to 1 mkm (**A**,**C**,**D**) and 200 nm (**B**,**E**).

The peptide R9F2 also led to diverse morphological patterns of *C. albicans* cells during 75 min, similar to peptide (KFF)3K. Both slightly changed cells (Figure 9D) and cells with destroyed organoids were observed in sections (Figure 9A,C). In many cells, a vacuole acquired electron-transparent content (Figure 9C), which, obviously, reflects a violation of the water-ion balance in damaged cells in this period. Deep invaginations of plasma membranes were observed in the cells (Figure 9B,D), probably reflecting the “development” of invaginations found earlier (Figure 7). Osmiophilic multimembrane (myelin-like) bodies were present in the cytoplasm (Figure 9A,C,E). These can be a derivative of plasma membrane folds described above, which became osmiophilic due to developing destructive processes. Formation of the same structures in mammalian cells treated with polyarginine peptides as CPP was described. The authors showed direct translocation of the peptide-transported molecule through plasma membranes and the ability of cells to eject multimembrane bodies outside [61]. Formation of multimembrane structures as a result of the interaction of polyarginine (R9) CPP with cell membranes and liposomes was reported in another study [59]. The formation of multimembrane structures from plasma membranes in *C. albicans* cells under R9F2 peptide influence observed in our work suggests a similar mechanism for penetration of the peptide into cells, despite differences in the molecular composition of the mammalian and fungal plasma membrane. On the surface of many cells wide osmiophilic regions of high electron density were seen (Figure 9A,C), the structural details of which were poorly distinguished even without section contrasting. We think that these regions may form from the extended folds (Figure 7A) during destructive processes resulting in osmiophilia of plasma membranes.

A six-hour incubation of *C. albicans* with peptide (KFF)3K led to an increase of dead cells (Figure 8F), the cytoplasm of which contained shapeless amorphous and granular material, membrane fragments, lipid droplets, and myelin-like structures—the “remains” of organoids. The rigid cell wall provided the visibility of cell integrity even after complete organoid destruction (Figure S2). Individual cells with a “normal” morphology were noted in sections in this period. Incubation of *C. albicans* with peptide R9F2 for 6 h also led to cell destruction; the cell wall limited the accumulation of detritus (Figure S2). Cells showing “normal” morphology and budding patterns were almost absent in the sections. 

To determine the specificity of observed structural changes in *C. albicans* when exposed to peptides (KFF)3K and R9F2, we analyzed fungal cells exposed to chlorhexidine. It turned out that the structural changes caused by this antiseptic (Figure S3) were completely different from the changes induced by the peptides, which once again confirms the specificity and depth of the observed changes in the morphology of fungal cells upon exposure to peptides.

## 4. Conclusions

Currently, direct methods for observing the molecular interactions of cell structures with compounds attacking the cell do not exist, despite the impressive advances in the development of imaging methods. The use of fluorescent labeling is the most common method of research in modern cell biology. However, it is appropriate to recall that attachment of any label to the molecule under study could change its properties and therefore can distort the processes of interaction with the cell. Modern TEM allows identifying cell structures and analyzing their changes, as well as reconstructing dynamics of these changes using a series of samples collected in the course of a study.

This study showed the antifungal effect of peptides (KFF)3K and R9F2 on *C. albicans* cells in the logarithmic growth phase (MIC of 20 and 10 μM, respectively) and suppression of hyphal growth. Studying the dynamics of changes in *C. albicans* ultrastructure under the influence of peptides (KFF)3K and R9F2 established differences in the temporal and structural characteristics of the effect of peptides, evidently related to their physicochemical properties. The first target for both peptides was the plasma membrane, and is its “alignment” was shared morphological manifestation of their effect, while the detection time of ultrastructural changes differed; effects of the R9F2 peptide lagged behind those of the (KFF)3K peptide.

Undoubtedly, morphological changes in cells occur later than molecular interactions, which caused these changes. However, the time of their registration is not as important as the observed differences in the manifestation of peptide’s effects. The appearance of osmiophilic sites in plasma membranes of *C. albicans* cells upon incubation with peptide (KFF)3K preceded morphological changes in cell walls by at least 15 min. This suggests that the peptide quickly overcomes the cell wall, and this process is not visible at the ultrastructure level. Peptide R9F2 also overcomes the cell wall without visible changes; herewith, the peptide-induced changes in plasma membranes were recorded later and had a different nature. The initial morphologic manifestations of the peptides’ effect were distinctly different, and the impact of peptide (KFF)3K on plasma membranes appeared more destructive. Vacuole changes in *C. albicans* cells under peptide (KFF)3K action were recorded simultaneously with changes in plasma membranes, and their nature indicates a change in the cell water-ion balance. The damage to plasma membranes observed at the same time suggests the possibility of uncontrolled migration of water and ions into/from the cytoplasm, which was reflected in the main “operator” of the water homeostasis system, i.e., vacuole. Preservation of the “normal” structure of *C. albicans* cells for at least 30 min when exposed to the R9F2 peptide once again indicated differences in the mechanisms of the influence of the two peptides on cells. Osmiophilic multimembrane structures that appeared over time indicate destruction processes of plasma membranes. Obviously, TEM was unable to recognize the nature of chemical modifications unfolding in a cell under the influence of a preparation. However, any morphological change signals a chemical one and importantly, indicates its location. 

We used *C. albicans* in the logarithmic phase of growth, when the cells are at different stages of the life cycle, and this allowed us to see how different the morphological manifestations of experimental effects can be, reflecting characteristics of cell reactions to the treatment. Unfortunately, it is impossible to compare the data we obtained with published values, not only because we studied dynamics of ultrastructural changes, but because other researchers use different methods and different experimental designs. Nevertheless, we believe that the data presented by us will be useful for researchers working on the development of new antifungals, since the presented experimental methodology allows observation of the dynamics of fungal cell development disorders and identification of the probable mechanisms of antimicrobial effects of the studied preparations.

## Supplementary Materials

The following are available online at https://www.mdpi.com/2076-2607/8/4/582/s1, Figure S1: Destroyed *C. albicans* cells after 6 h of incubation with peptides (KF)3K and R9F2. Figure S2: Variable morphology of *C. albicans* destroyed cells after 6 h of incubation with peptides (KF)3K and R9F2. Figure S3: Ultrastructure of *C. albicans* cells treated with chlorhexidine. Table S1: Minimum inhibitory and fungicidal concentrations of drugs for species of the genus *Candida.* Table S2: Influence of R9F2 and (KFF)3K peptides on size and shape of *C. albicans* cells.
