# Supplementary material for: Changes in the Ultrastructure of Candida albicans Treated with Cationic Peptides
Source: Microorganisms. 2020 Apr 17;8(4):582. doi: 10.3390/microorganisms8040582 (PMC7232200; doi:10.3390/microorganisms8040582)
Supplement: Supplementary file 1 [file microorganisms-08-00582-s001.pdf]

Supplementary materials

For article “Changes of ultrastructure of *Candida albicans* treated with cationic peptides”, by Grigor’eva, A. et al.

Table S1. Minimum inhibitory and fungicidal concentrations of drugs for species of the genus *Candida*

Species and strains	Preparation	MIC* (μM)	MFC** (μM)
C. albicans, strain CEMTC***- 34	R9F2	10	>10
	(KFF)3K	20	20
	Chlorhexidine	12.5	25
C. albicans, strain ATCC 10231	R9F2	10	>10
	(KFF)3K	20	20
	Chlorhexidine	12.5	25
C. albicans, strain 1550	R9F2	10	10
	(KFF)3K	10	20
	Chlorhexidine	12.5	12.5
C. parapsilosis, strain CEMTC 2529	R9F2	10	>10
	(KFF)3K	20	>20
	Chlorhexidine	25	50
C. glabrata, strain CEMTC 2563	R9F2	>10	>10
	(KFF)3K	>20	>20
	Chlorhexidine	12.5	12.5
C. tropicalis, strain CEMTC 2385	R9F2	10	>10
	(KFF)3K	10	>20
	Chlorhexidine	25	50
C. guilliermondii, strain CEMTC 1246	R9F2	10	10
	(KFF)3K	5	10
	Chlorhexidine	6.3	12.5

*Minimum inhibitory concentration – the lowest concentration of the antibiotic that inhibits in vitro visible growth of the culture

** The minimum fungicidal concentration is the lowest concentration of an antibiotic that causes the death of 99.9% of the initial number of culture cells in vitro.

*** Collection of Extremophile Microorganisms and Type Cultures of ICBFM SB RAS.

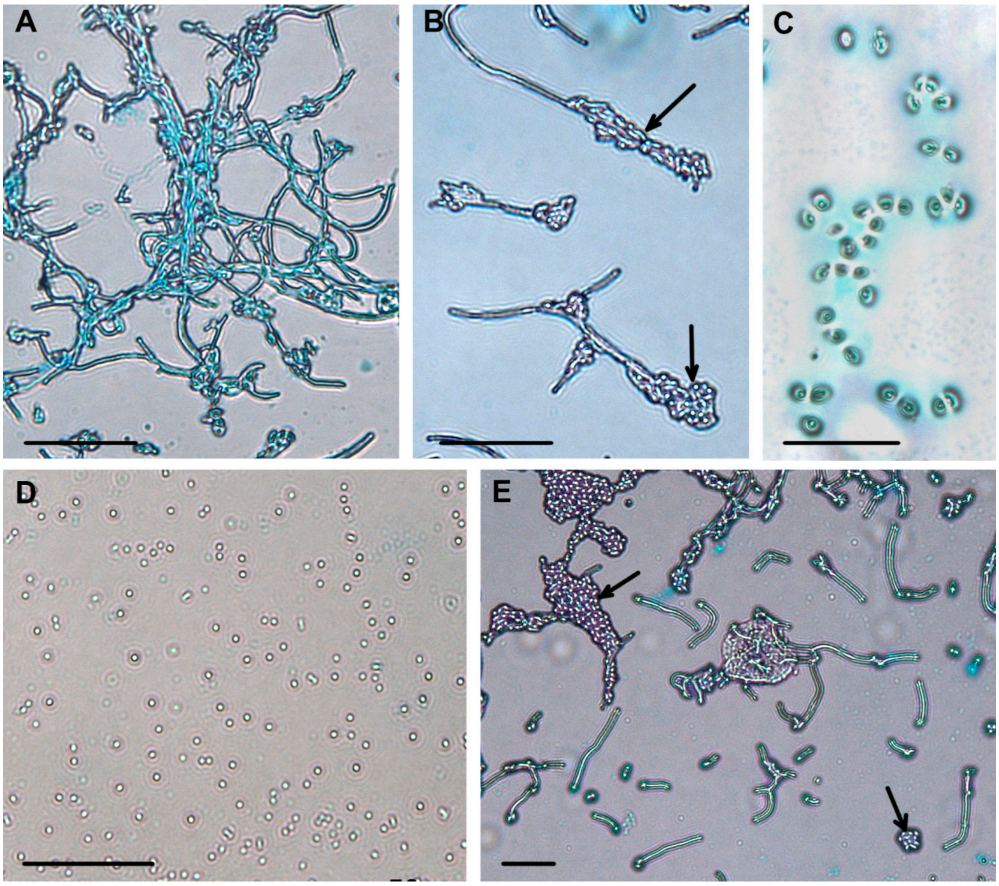

Figure S1. Hiphae of intact *C. albicans* culture (**A**). Clusters of spherical C. albicans cells (are shown by arrows) after incubation with 2.5 μM KFF)3K peptide (**B**). Spherical cells of C. albicans after incubation with 5 μM (KFF)3K peptide (**C**) and R9F2 peptide (**D**). Clusters of spherical C. albicans cells (are shown by arrows) after incubation with 2.5 μM chlorhexidine (**E**). Light microscopy, smears. Azure-II stain. Scale bars correspond to 50 μm.

Table S2. Influence of R9F2 and (KFF)3K peptides on size and shape of C. albicans cells

Incubation time	Peptide	Length, mean±SE	Width, mean±SE	Morphologic index (Length\width), mean±SE
30 min – 24 h	**Intact cells**	**3.9±0.10**	**3.4±0.09**	**1.12±0.02**
30 min	R9F2	4.5±0.21**	3.5±0.15	1.27±0.05**
30 min	(KFF)3K	4.7±0.16***	3.6±0.07	1.32±0.04***
45 min	R9F2	4.8±0.23***	3.7±0.09	1.32±0.06***
45 min	(KFF)3K	4.2±0.14*^	3.5±0.08	1.24±0.05*
75 min	R9F2	4.7±0.16***	3.5±0.11	1.36±0.05***
75 min	(KFF)3K	4.3±0.15*	3.3±0.16	1.36±0.06***
105 min	R9F2	4.5±0.12***	3.7±0.09	1.23±0.04*
105 min	(KFF)3K	4.4±0.13***	3.2±0.15***^	1.37±0.05*
2 h	R9F2	5.0±0.21***	3.7±0.14**	1.37±0.05***
2 h	(KFF)3K	4.8±0.25***	3.9±0.15*	1.23±0.04**^
4 h	R9F2	5.1±0.15***	4.2±0.11***	1.2±0.04***
4 h	(KFF)3K	5.2±0.18***	3.8±0.09**^	1.4±0.05***

The size of intact cells and cells incubated with R9F2 and (KFF)3K peptides, were measured on semithin sections. Each value represents a mean of measurements of 50-60 individual cells.

The following marks designate significance of differences (t-test):

*** - $P < 0.001$, ** - $P < 0.01$, * - $P < 0.05$ the peptides from **intact cells;**

^^ - $P < 0.01$, ^ - $P < 0.05$ (KFF)3K peptide from R9F2 peptide.

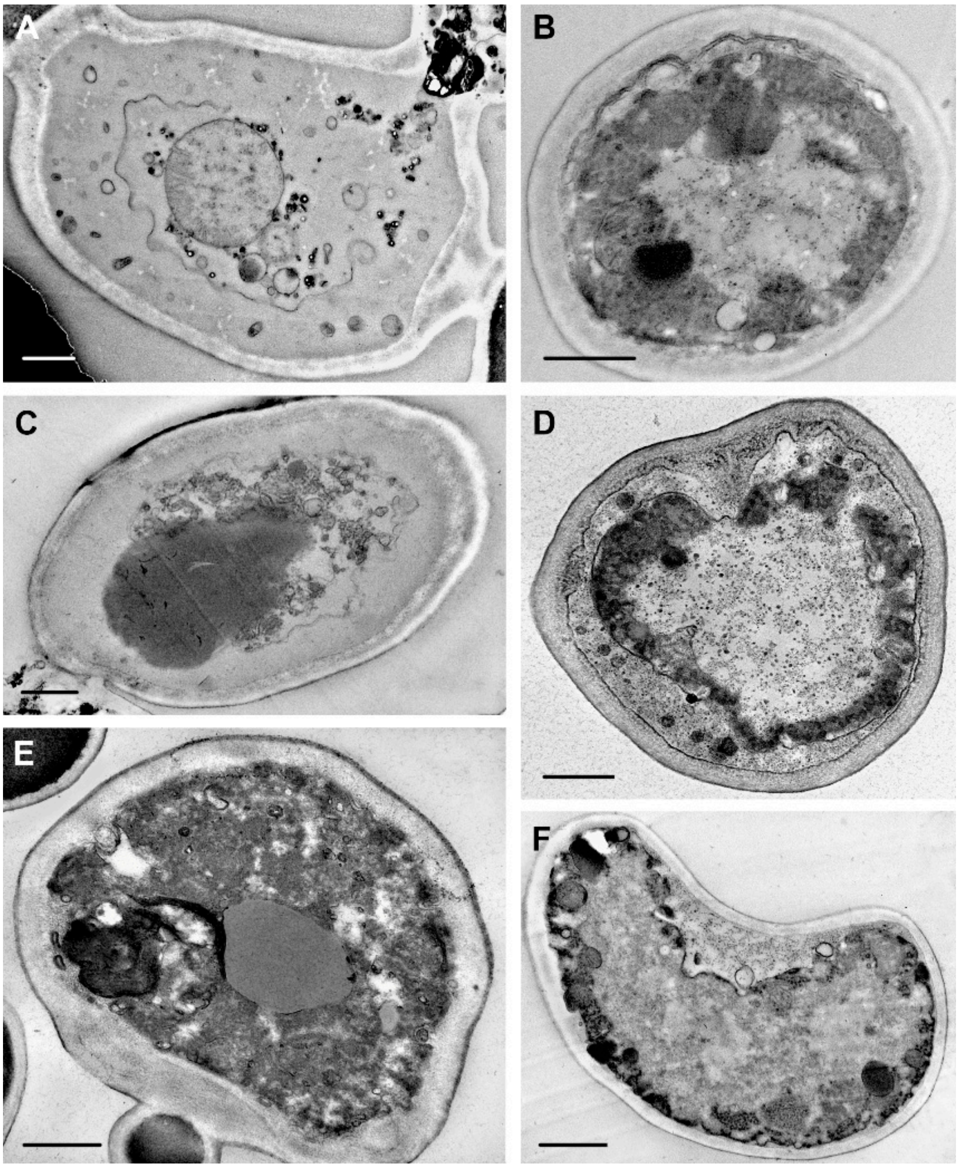

Figure S2. Variable morphology of *C. albicans* destroyed cells after 6 h of incubation with peptides (KF)3K (A, C, E) and R9F2 (B, D, F). A, B – without contrasting. Scale bars correspond to 2 μm.

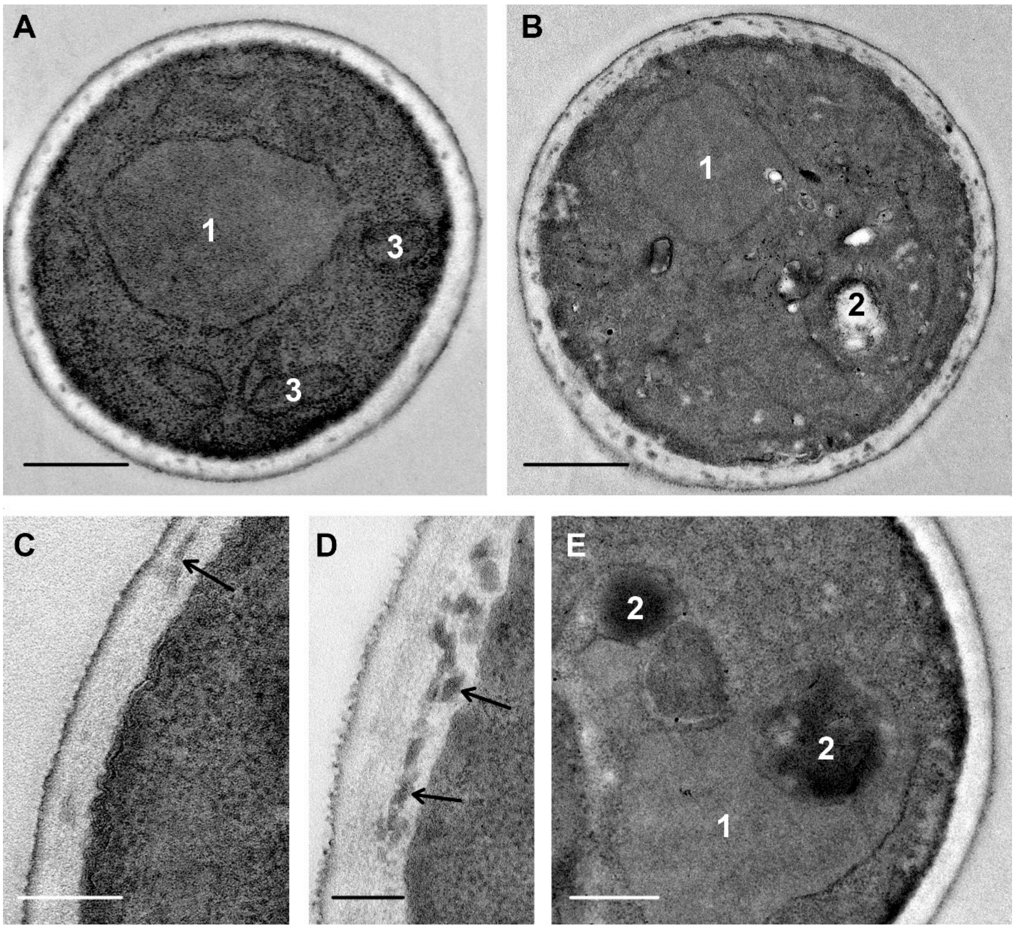

Figure S3. Ultrastructure of *C. albicans* cells treated with chlorhexidine for 45 min (**A**) and 75 min (**D-E**). Less (**A**) and more pronounced (**B**) cell damage. Early (**C**) and late alterations (**D)** of the cell wall structure. **E** – Part of a cell showing vacuole. **1** – nucleus; **2** – vacuole; **3** – mitochondria. Arrows show electron dense material inside cell wall. Scale bars correspond to 1 μm (A, B), 200 nm (C, D) and 500 nm (E).
